# DSCR9/miR-21-5p axis inhibits pancreatic cancer proliferation and resistance to gemcitabine via BTG2 signaling: DSCR9/miR-21-5p/BTG2 regulates pancreatic cancer

**DOI:** 10.3724/abbs.2022194

**Published:** 2022-12-26

**Authors:** Hui Huang, Xia Li, Xianlin Zhang, Zhiqiang Li, Duo Han, Wenzhe Gao, Ling Liu, Cheng Peng, Hongwei Zhu, Xiao Yu

**Affiliations:** 1 Department of Hepatopancreatobiliary Surgery Third Xiangya Hospital Central South University Changsha 410013 China; 2 Department of Endocrinology Third Xiangya Hospital Central South University Changsha 410013 China; 3 Department of General Surgery Affiliated Renhe Hospital of China Three Gorges University Yichang 443001 China; 4 Department of Cardiology Second People’s Hospital of Hunan Province Hunan University of Chinese Medicine Changsha 410007 China

**Keywords:** pancreatic cancer, lncRNA DSCR9, miR-21-5p, BTG2, proliferation, gemcitabine resistance

## Abstract

The outcome of pancreatic adenocarcinoma (PAAD) patients is poor, given resistance to gemcitabine. Long noncoding RNA (lncRNA) has been implicated in the carcinogenesis of pancreatic cancer; however, its function and mechanism in PAAD resistance to gemcitabine (GEM) are yet unknown. Herein, we demonstrate that lncRNA DSCR9 is significantly reduced in PAAD *in vitro* and *in vivo*. CCK-8, BrdU and flow cytometry assays show that overexpression of DSCR9 markedly suppresses pancreatic cancer cell proliferation and invasion, and promotes apoptosis under gemcitabine treatment. BTG2 acts as a tumor suppressor by reducing the proliferation and invasion of pancreatic cancer cells and increasing gemcitabine-induced apoptosis. Immunofluorescence (IF) staining combined with fluorescence *in situ* hybridization (FISH) of pancreatic cancer tissues shows that DSCR9 and BTG2 are both increased in pancreatic cancer tissues. Luciferase assay shows that miR-21-5p simultaneously binds to DSCR9 and 3′UTR of BTG2; DSCR9 relieves miR-21-5p-induced inhibition of BTG2 by competing with BTG2 for miR-21-5p binding. Overexpression of miR-21-5p enhances the invasiveness of pancreatic cancer cells by promoting cancer cell proliferation and invasion and attenuating gemcitabine-induced apoptosis. Overexpression of miR-21-5p attenuates the effect of DSCR9 overexpression on BTG2 expression and invasiveness of pancreatic cancer cells. Finally, miR-21-5p expression is increased, while BTG2 expression is decreased in pancreatic cancer tissues. miR-21-5p is negatively correlated with DSCR9 and BTG2. In conclusion, the DSCR9/miR-21-5p/BTG2 axis modulates pancreatic cancer proliferation, invasion, and gemcitabine resistance.

## Introduction

Pancreatic adenocarcinoma (PAAD), a digestive system malignancy, remains a challenge to public health due to its extremely high global incidence and mortality. Furthermore, approximately 90% of pancreatic cancer patients suffer from distant metastasis when first diagnosed, and only 20% of patients are suitable for surgery [ 1‒ 4] . In recent decades, gemcitabine has been used as a first-line agent for chemotherapy in patients with advanced PAAD. However, the acquisition of drug resistance has posed a significant limitation to the clinical efficacy of gemcitabine [2]. The key to improving the prognosis of PAAD patients is to investigate the intrinsic mechanisms of pancreatic cancer invasion and novel molecular therapeutic targets for reversing drug resistance.

Long noncoding RNAs (lncRNAs) are RNAs defined as transcripts with lengths exceeding 200 nucleotides that are not translated into proteins. As previously reported, lncRNAs can function as tumor suppressors or oncogenic lncRNAs and play important roles in the biological behaviors of cancer cells, including cell proliferation, apoptosis, migration, invasion, autophagy, metabolism, senescence, and differentiation. In addition, it has been revealed that lncRNA deregulation contributes to drug resistance in tumors, including colorectal carcinoma, hepatocellular carcinoma (HCC), breast carcinoma, and pancreatic cancer [ 5‒ 8] . Several lncRNAs have been reported to serve as oncogenic or anti-cancerous factors in PAAD. By performing integrative bioinformatics analyses, Qi *et al*. [9] identified a signature consisting of 8 immune-related lncRNAs as a potential predictor for the survival of PAAD patients. LINC01232 facilitates the mRNA stability of its nearby gene transmembrane 9 superfamily member 2 (TM9SF2), therefore acting as an oncogenic factor in PAAD [10]. However, the role and mechanism of lncRNA in mediating PAAD chemoresistance remain unknown.

In addition to lncRNAs, miRNAs have also been identified as important regulators in a variety of tumors, acting as tumor oncogenes or tumor suppressors to modulate cancer cell phenotypes [ 11, 12] . In PAAD, several miRNAs have been demonstrated as prognostic/diagnostic markers [ 13‒ 15] , while miR-32, miR-320b, and miR-340-5p have been reported as anti-tumor miRNAs [ 16‒ 18] . Notably, there is increasing evidence that lncRNAs can competitively bind to miRNAs via their miRNA response elements (MREs) to act as competing endogenous RNAs (ceRNAs), thereby modulating the expression of target RNAs [19]. Mechanistically, although the functional mechanisms of lncRNAs are complicated, the ceRNA mode of action has been regarded as the major way for lncRNAs to modulate the expressions of target miRNA/mRNA axes [19]. The lncRNA-miRNA ceRNA network has also been reported to play a role in PAAD prognosis [20]. Meanwhile, lncRNA/miRNA axes could modulate PAAD cancer cell phenotypes [ 18, 21, 22] . However, whether lncRNA could affect PAAD chemoresistance through the ceRNA mechanism is unclear.

In this study, we found that lncRNA DSCR9 is downregulated in pancreatic cancer, and can modulate pancreatic cancer cell proliferation, invasion, and resistance to gemcitabine through lncRNA/miRNA/mRNA axis.

## Materials and Methods

### Clinical sampling

A total of 15 paired pancreatic cancer tissues and adjacent noncancerous tissues were obtained from patients diagnosed with pancreatic cancer who underwent surgical resection at the Hepatobiliary and Pancreatic Surgery Department in the Third Xiangya Hospital of Central South University. All samples were formalin-fixed and paraffin-embedded. All of the experimental procedures were performed under the approval of the Ethics Committee of the University. Informed consent was signed by each patient enrolled. The clinical characters of patients are listed in Supplementary Table S1.

### Cell lines

The pancreatic cancer cell lines PANC-1 (CRL-1469; ATCC, Manassas, USA) and MIAPaCa-2 (CRM-CRL-1420; ATCC) were cultured in Dulbecco’s modified Eagle’s medium (30-2002; ATCC) supplemented with 10% FBS (Sigma, St Louis, USA). BxPC-3 (CRL-1687; ATCC) and AsPC-1 (CRL-1682; ATCC) cell lines were cultured in RPMI-1640 medium (30-2001; ATCC) supplemented with 10% FBS. Human pancreatic nestin-expressing cell line, hTERT-HPNE (CRL-4023; ATCC), was cultured in a mixture of 75% DMEM without glucose (D-5030; with an additional 2 mM L-glutamine and 1.5 g/L sodium bicarbonate; Sigma) and 25% Medium M3 Base (M300F-500; Incell Corp., San Antonio, USA) supplemented with 10% FBS (Sigma). All cells were cultured at 37°C in a 5% CO _2_ incubator.

### Bioinformatics analysis

To select differentially expressed genes (DEGs) in pancreatic ductal adenocarcinoma, the Gene Expression Omnibus (GEO) datasets GSE15471, GSE62452, and GSE28735 were downloaded using the R language GEOquery package ( https://www.ncbi.nlm.nih.gov/gds/?term=) [23], and the DEGs were analyzed by Limma package [24]. The selection threshold was set as |logFC|>0.40, adj.p.val<0.0001. Based on the Pan-cancer database in KMplot ( http://kmplot.com/), TCGA-PAAD database ( https://xenabrowser.net), and GSE78229, the association between lncRNA expression and pancreatic carcinoma patient overall survival was analyzed by the Cox-proportional hazards model (CoxPH). The Pearson correlation coefficient (R language Psych package [25]) was applied to analyze the coexpressed genes with DSCR9 in TCGA-PAAD. The coexpressed genes were applied for Gene Set Enrichment Analysis (GSEA) with R language clusterprofiler package [26].

### Cell transfection

lncRNA DSCR9 overexpression or BTG2 overexpression was generated in target cells by infecting lentivirus containing DSCR9 or BTG2, namely, lv-DSCR9 and lv-BTG2, synthesized by GenePharma (Shanghai, China). miR-21-5p overexpression or inhibition was generated in target cells by transfecting miR-21-5p mimics or inhibitor synthesized by GenePharma. Following the protocols, the above-described lentivirus or miRNAs were transfected into target cells with the help of polybrene or Lipofectamine 3000 (Invitrogen, Carlsbad, USA). The sequences for miRNA and plasmid construction are listed in Table 1.

**Table:** **Table 1** The sequences of primers and miRNAs used in this study

Target	Sequence (5′→3′)
RT-PCR
lncRNA DSCR9	F: CCCAAATGAAGCGAGGTGAAA R: ATGCGATGTGATGTGCTGTGC
β-actin	F: TTCCAGCCTTCCTTCCTGGG R: TTGCGCTCAGGAGGAGCAAT
miR-107	RT: GTCGTATCCAGTGCGTGTCGTGGAGTCGGCAATTGCACTGGATACGACTGATAG F: GAGCAGCATTGTACAGGG R: CAGTGCGTGTCGTGGA
miR-339-5p	RT: GTCGTATCCAGTGCGTGTCGTGGAGTCGGCAATTGCACTGGATACGACCGTGAG F: TCCCTGTCCTCCAGGAG R: CAGTGCGTGTCGTGGA
miR-21-5p	RT: GTCGTATCCAGTGCGTGTCGTGGAGTCGGCAATTGCACTGGATACGACTCAACA F: GCCGTAGCTTATCAGACTGA R: CAGTGCGTGTCGTGGA
BTG2	F: ACCACTGGTTTCCCGAAAAG R: CTGGCTGAGTCCGATCTGG
U6	F: CTCGCTTCGGCAGCACA R: AACGCTTCACGAATTTGCGT
Vector construction
DSCR9 overexpression	F: CTACCGGACTCAGATCTCGAGCTTCTACCCGGGGGCTCC R: GTACCGTCGACTGCAGAATTCTTTTTTTTTTTTTTTTTTTTTTTTTTTTTTTTTTTTTCTAAAACAATA
BTG2 overexpression	F: CTACCGGACTCAGATCTCGAGATGAGCCACGGGAAGGGA R: GTACCGTCGACTGCAGAATTCCTAGCTGGAGACTGCCATCACG
WT-DSCR9	F: AATTCTAGGCGATCGCTCGAGACTTCCCGGAAGAGCCCA R: ATTTTATTGCGGCCAGCGGCCGCCAGGCGTGGCTTCAAAAGC
Mut-DSCR9	F: ATTCTTGGCGCTGGATTGCCGCTTCTCTTTTTTATCACC R: CAATCCAGCGCCAAGAATAAATGATGACCGCCG
WT-BTG2 3′UTR	F: AATTCTAGGCGATCGCTCGAGTGTCTGCAAACAGGTCCCTGC R: ATTTTATTGCGGCCAGCGGCCGCTTTATCCTACTACATTTTTATTAAAGTAACAAAA
Mut-BTG2 3′UTR	F: GGTGGATGATTCTGACAGAAAAGACAAAGGTTACTAA R: CTGTCAGAATCTCCACAAACATACTACTACTATTTATTTTTACTTAATAGGA
NC mimics	F: UUCUCCGAACGUGUCACGUTT R: ACGUGACACGUUCGGAGAATT
NC inhibitor	F: CAGUACUUUUGUGUAGUACAA
miR-21-5p mimics	F: UAGCUUAUCAGACUGAUGUUGA R: AACAUCAGUCUGAUAAGCUAUU
miR-107 mimics	F: AGCAGCAUUGUACAGGGCUAUCA R: AUAGCCCUGUACAAUGCUGCUUU
miR-21-5p inhibitor	F: UCAACAUCAGUCUGAUAAGCUA
miR-107 inhibitor	F: UGAUAGCCCUGUACAAUGCUGCU
FISH DSC9 probe	GCCCATTCTTTCACCTCGCTTCATT

RT: reverse transcription primer, F: forward primer; R: reverse primer

### Real-time PCR

Total RNA was extracted, processed, and examined for target lncRNA, mRNA, and miRNA expression following the methods described previously [27]. The expression levels of lncRNA, mRNA, and miRNA were detected by using SYBR green PCR Master Mix (Qiagen, Hilden, Germany) using *β-actin* (for mRNA examination) or *RNU6B* (for miRNA examination) as an endogenous control. PCR reaction conditions is as follows: initial denaturation at 95°C for 5 min, followed by 40 cycles of denaturation at 95°C for 5 s, and annealing and extension at 60°C for 30 s. The data were processed using the 2 ^‒ΔΔCT^ method [28]. The primer sequences are listed in Table 1.

### Immunohistochemical (IHC) staining

Tissue samples were sectioned into 5 μm-thick slices, deparaffinized in xylene, and rehydrated in a series of graded alcohols. Antigen retrieval was performed by immersing the slides in sodium citrate. Endogenous peroxidase was blocked by a 10-min incubation with 3% H _2_O _2_. Next, the slices were incubated with primary antibody anti-BTG2 (ab85051; Abcam, Cambridge, UK) overnight at 4°C, washed three times with PBS, and incubated with horseradish peroxidase (HRP)-conjugated secondary antibody (ab205718; Abcam) for 30 min. Finally, immunostaining was performed using a diaminobenzidine (DAB) substrate kit (ab64238; Abcam). The sections were observed with a CKX41 optical microscope (Olympus, Tokyo, Japan).

### Immunofluorescence (IF) staining combined with fluorescence *in situ* hybridization (FISH)

After paraffin sections were dewaxed and incubated in a microwave oven for 10 min with citric acid antigen repair buffer (pH 6.0), the sections were washed three times with PBS (pH 7.4) and incubated in 3% hydrogen peroxide solution for 25 min at room temperature in the dark to block endogenous peroxidase. After washing, the sections were incubated with 10% goat serum at room temperature for 30 min. BTG2 antibody prepared in PBS (dilution ratio 1:1000) was added to the sections, and the sections were incubated in a wet box at 4°C overnight. A secondary antibody (HRP-labeled) of the corresponding species was added and incubated for 50 min at room temperature. Sections were incubated with a membrane-breaking solution for 10 min at room temperature to allow cell permeation. Sections were further incubated with diluted TSA-520 for 20 min at 37°C. Later, after antigen repair, the proteinase K working solution (10 μg/mL) was added to the sections and incubated for 5 min at 37°C. The sections were prehybridized by diluting salmonid sperm DNA with hybridization buffer and incubating at 37°C for 60 min. Probe DSCR9 labeled with Cy3 was diluted with hybridization buffer (1:100), and sections were hybridized overnight in a constant temperature incubator at 42°C. Sections were washed with a gradient concentration of SSC liquid, the nuclei were stained with DAPI, and sections were sealed with an anti-fluorescence quenching sealer and observed with a CKX53 fluorescence microscope (Olympus). Green fluorescence indicates BTG2 expression. Red fluorescence indicates DSCR9 expression.

### Western blot analysis

Total protein was extracted from cells and tissues using RIPA lysis buffer (Beyotime, Shanghai, China). Then, 50 μg protein were separated by 10%‒15% SDS-PAGE and transferred onto polyvinylidene fluoride (PVDF) membranes. Nonspecific binding was blocked by incubation with 5% nonfat dry milk in Tris-buffered saline Tween (TBST) for 2 h. After that, the membranes were probed with the appropriate primary antibodies at 4°C overnight, followed by another incubation with the corresponding secondary antibodies for 2 h at room temperature. The primary antibodies used were as follows: anti-Ki-67 (ab15580; Abcam), anti-PCNA (ab29; Abcam), anti-BTG2 (ab85051; Abcam), and anti-β-actin (6008-1-Ig; Proteintech). Actin was used an endogenous control. The immunoreactive proteins were visualized and examined using an enhanced chemiluminescence reagent (BeyoECL Star Kit; Beyotime).

### Cell viability assay

Cell viability was examined using a CCK-8 kit (Beyotime). After transfection or treatment, cells were seeded into 96-well plates at a density of 5×10 ^3^ cells/well. Two hours before the examination, 20 μLof CCK-8 solution was added to each well, followed by incubation at 37°C. The optical density (OD) value was determined at a wavelength of 490 nmwith a VICTOR NIVO microplate reader (PerkinElmer, Norwalk, USA).

### BrdU assay

DNA synthesis was examined by a colorimetric immunoassay based on the measurement of 5-bromo-2′-deoxyuridine (BrdU) incorporation during DNA synthesis. After 24 h of incubation, the cells were labeled with BrdU for 3 h at 37°C. Cells were fixed and incubated with a peroxidase-conjugated anti-BrdU antibody. Then, the peroxidase substrate 3,3′,5,5′-tetramethylbenzidine was added, and BrdU incorporation was quantified with the VICTOR NIVO microplate reader at 450 nm.

### Cell invasion assay

Cells (5×10 ^5^) were plated on the top side of polycarbonate Transwell (Corning Co, Corning, USA) filters coated with Matrigel (Becton, Dickinson and Company, Franklin Lakes, USA) for invasion examination. For Transwell invasion assay, cells were suspended in medium without serum, and medium without serum was used in the bottom chamber. The cells were incubated at 37°C for 48 h. The noninvasive cells in the top chambers were removed with cotton swabs. The invaded cells on the lower membrane surface were fixed in 100% methanol for 10 min, air-dried, stained with crystal violet solution, and then counted under an optical microscope.

### Cell apoptosis assay

The chemosensitivity of cells to gemcitabine was tested by cell apoptosis assay. Briefly, cells were treated with 10 μM gemcitabine for 48 hand then digested by trypsin and collected. Cells were resuspended in 500 μL binding buffer and incubated with 5 μL Annexin V-FITC and 5 μL propidium iodide (PI) at room temperature in the dark for 15 min. Then, the cell apoptosis were analyzed by flow cytometry on the Agilent NovoCyte flow cytometer (Agilent Technologies, Palo Alto, USA).

### Luciferase reporter assay

To test the binding between miR-21-5p and BTG2 3′UTR or lncRNA DSCR9, the wild-type or mutated BTG2 3′UTR or DSCR9 was cloned to the downstream of the Renilla psiCHECK2 vector (Promega, Madison, USA), named wt-BTG2 3′UTR/wt-DSCR9 or mut-BTG2 3′UTR/mut-DSCR9. Next, 293T cells (CRL-3216; ATCC) were cotransfected with two types of luciferase reporter vectors and miR-21-5p mimics/miR-21-5p inhibitor. Luciferase activity was examined using the Dual-Luciferase Reporter Assay System (Promega, Madison, USA).

### Statistical analysis

The data were analyzed with GraphPad software and are expressed as the mean±standard deviation (SD). Among-group and intragroup data comparisons were performed by ANOVA and Student’s *t* tests. *P*<0.05 indicates a statistically significant difference.

## Results

### lncRNAs deregulated in pancreatic cancer and associated with cancer progression

**Figure 1:**
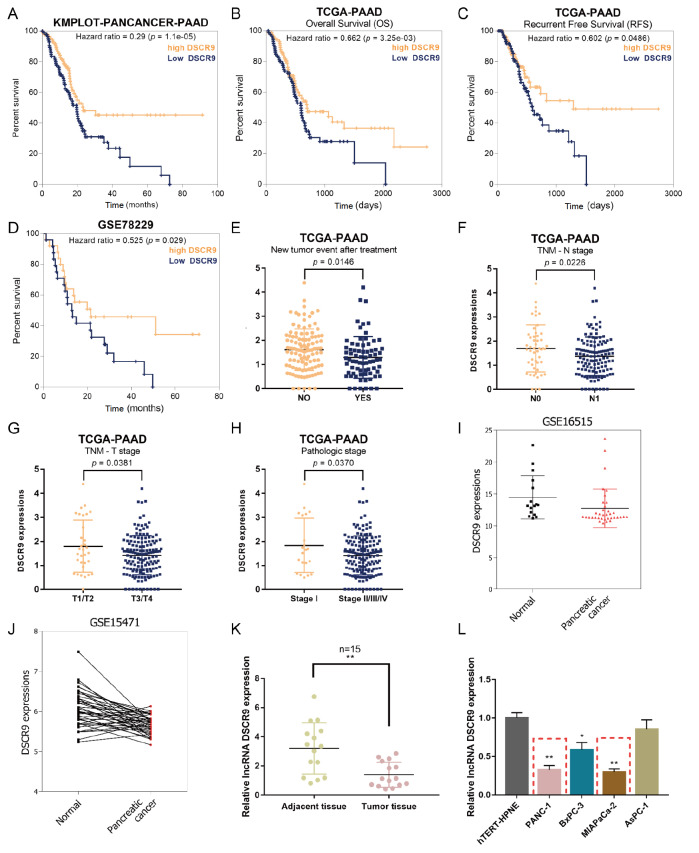
Figure 1 Expression of lncRNA DSCR9 and its correlation with pancreatic cancer prognosis (A) Subjects with pancreatic cancer from the Pan-cancer database in KMplot ( http://kmplot.com/) were divided into a high DSCR9 expression group and a low DSCR9 expression group. The correlation of DSCR9 expression with the survival percentage in subjects with pancreatic cancer was analyzed using the Cox proportional hazards (CoxPH) model. (B) Subjects with pancreatic cancer from the TCGA-PAAD database were divided into a high DSCR9 expression group and a low DSCR9 expression group. The correlation of DSCR9 expression with overall survival (OS) in subjects with pancreatic cancer was analyzed using CoxPH model. (C) Subjects with pancreatic cancer from the TCGA-PAAD database were divided into a high DSCR9 expression group and a low DSCR9 expression group. The correlation of DSCR9 expression with recurrence-free survival (RFS) in subjects with pancreatic cancer was analyzed using the CoxPH model. (D) Subjects with pancreatic cancer from GSE78229 were divided into a high DSCR9 expression group and a low DSCR9 expression group. The correlation of DSCR9 expression with the survival percentage in subjects with pancreatic cancer was analyzed using the CoxPH model. (E) DSCR9 expression in patients with or without relapse after initial treatment according to TCGA-PAAD database. (F) DSCR9 expression in patients in N0 or N1 stage (TNM staging system; with regional lymph node metastasis) according to TCGA-PAAD database. (G) DSCR9 expression in patients in T3/T4 or T1/T2 stages (TNM staging system) according to TCGA-PAAD database. (H) DSCR9 expression in patients with stage I or stage II/III/IV disease (AJCC WHO pathological grading) according to the TCGA-PAAD database. (I) DSCR9 expression in 36 pancreatic cancer tissues and 16 noncancerous tissues according to GSE16515. (J) DSCR9 expression in 40 paired pancreatic cancer and noncancerous tissues according to GSE15471. (K) DSCR9 expression was examined in 15 paired collected pancreatic cancer and noncancerous tissues by real-time PCR. (L) DSCR9 expression was examined in the human pancreatic nestin-expressing cell line hTERT-HPNE and four pancreatic cancer cell lines PANC-1, BxPC-3, MIAPaCa-2, and AsPC-1 by real-time PCR. * P<0.05, ** P<0.01.

To select differentially expressed genes, we performed bioinformatics analysis by downloading three sets of pancreatic cancer chips (GSE15471, GSE62452, and GSE28735) from the Gene Expression Omnibus (GEO). A total of 1768 genes were found to be significantly downregulated in pancreatic cancer samples (|logFC|>0.40, adj.p.val<0.0001). Hierarchical clustering results from three sets of expression profiles exhibited systematic alterations in the levels of mRNAs, including lncRNAs, between normal and pancreatic cancer tissues ( Supplementary Figure S1A,C,E), while the volcano plot diagram in Supplementary Figure S1B,D,F showed the distribution of differentially expressed mRNAs, including lncRNAs, between noncancerous and pancreatic carcinoma tissue samples. lncRNAs were labeled by their names in the volcano plot diagram. Next, the downregulated lncRNAs (LINC01128, DGCR11, DSCR9, LINC00339, LINC00671, TDH, and ALMS1P1) overlapped in the three GSE datasets were selected, and the association between the expressions of lncRNAs and the survival rate of pancreatic carcinoma patients was evaluated by KMplot ( Figure 1A and Supplementary Figure S2A‒G). Among them, lncRNA DSCR9 showed the lowest Hazard Ratio (HR, 0.29, *P*<0.01), suggesting that DSCR9 might be a protective factor in pancreatic cancer patients.

### Expression of lncRNA DSCR9 and its correlation with pancreatic cancer prognosis

Before investigating the specific effects of DSCR9 on pancreatic cancer cell phenotypes, we further explored the correlation of DSCR9 expression with the prognosis of patients with pancreatic cancer based on TCGA-PAAD database. The CoxPH model showed that higher DSCR9 expression was correlated with better overall survival ( Figure 1B) and better recurrence-free survival (RFS) ( Figure 1C) in subjects with pancreatic cancer. According to GSE78229, the CoxPH model shows that higher DSCR9 expression correlates with a higher survival percentage ( Figure 1D).

Meanwhile, in the TCGA-PAAD database, DSCR9 expression was significantly downregulated in patients with relapse after initial treatment compared to that in patients without relapse ( Figure 1E). Patients in the N1 stage (TNM staging system; with regional lymph node metastasis) had significantly lower DSCR9 expression than those in the N0 stage (without regional lymph node metastasis) ( Figure 1F). DSCR9 expression was significantly lower in patients in advanced T3/T4 stages (TNM staging system) than in patients in the early T1/T2 stages ( Figure 1G). DSCR9 expression was significantly downregulated in patients in advanced stage II/III/IV (AJCC WHO pathological grading) compared to that in patients in the early-stage I ( Figure 1H). In addition, based on the microarray expression profile GSE16515, DSCR9 expression was also significantly downregulated in 36 pancreatic carcinoma tissue samples compared with that in 16 noncancerous tissue samples ( Figure 1I). DSCR9 expression was significantly downregulated in 40 pancreatic carcinoma tissue samples compared with that in 40 paired noncancerous tissue samples ( Figure 1J).

Next, we examined DSCR9 expression in 15 paired collected pancreatic carcinoma and noncancerous tissue samples. The expression of DSCR9 was dramatically decreased in tumor tissue samples ( Figure 1K). DSCR9 expression was also significantly downregulated in four pancreatic carcinoma cell lines, PANC-1, BxPC-3, MIAPaCa-2, and AsPC-1, compared to that in the human pancreatic nestin-expressing cell line hTERT-HPNE ( Figure 1L). DSCR9 expression was much lower in PANC-1 and MIAPaCa-2 cell lines; thus, we selected PANC-1 and MIAPaCa-2 cell lines for further experiments.

### Specific effects of DSCR9 on pancreatic carcinoma cell proliferation, invasion, and gemcitabine resistance

**Figure 2:**
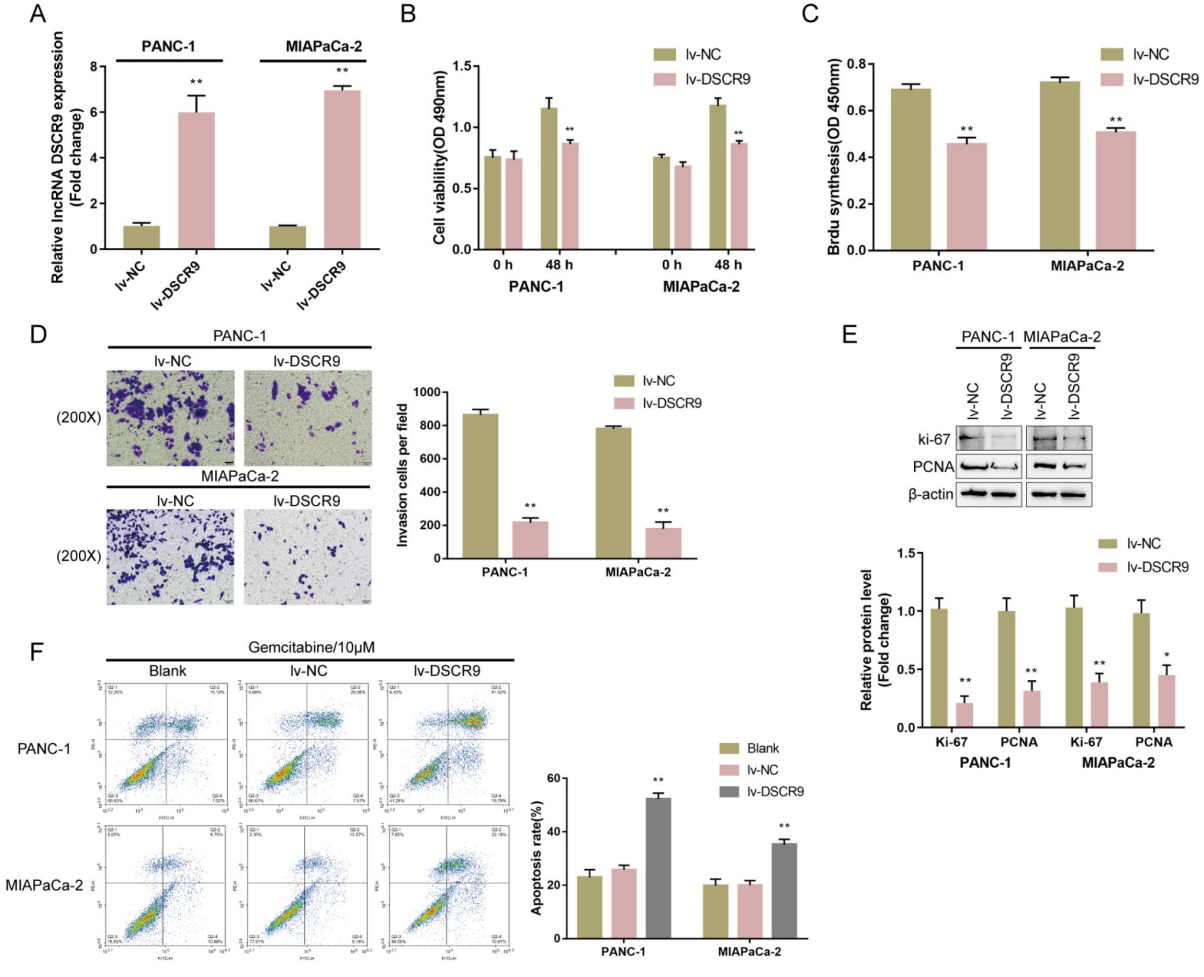
Figure 2 Specific effects of DSCR9 on pancreatic cancer cell proliferation, invasion, and gemcitabine resistance (A) DSCR9 overexpression was generated in PANC-1 and MIAPaCa-2 cells by infection with lv-DSCR9. The infection efficiency was confirmed by real-time PCR. PANC-1 and MIAPaCa-2 cells were infected with lv-NC (negative control)/lv-DSCR9 and examined for (B) cell viability by CCK-8 assay; (C) DNA synthesis capacity by BrdU assay; (D) cell invasion by Transwell assay; and (E) the protein levels of Ki-67 and PCNA by western blot analysis. (F) Noninfected, lv-NC-infected, or lv-DSCR9-infected PANC-1 and MIAPaCa-2 cells were treated with gemcitabine (10 μM) for 48 h and examined for cell apoptosis by flow cytometry. * P<0.05, ** P<0.01.

To investigate the specific effects of DSCR9 *in vitro*, we overexpressed DSCR9 in PANC-1 and MIAPaCa-2 cell lines by infecting them with lv-DSCR9. The infection efficiency was confirmed by real-time PCR ( Figure 2A). Next, PANC-1 and MIAPaCa-2 cell lines were infected with lv-DSCR9 and examined for related indexes. In both cell lines, DSCR9 overexpression significantly inhibited cell viability ( Figure 2B), suppressed DNA synthesis capacity ( Figure 2C), and inhibited cell invasion ( Figure 2D). Consistently, Ki-67 and PCNA protein levels were remarkably reduced in both cell lines by DSCR9 overexpression ( Figure 2E). To validate the role of DSCR9 in pancreatic carcinoma cell resistance to gemcitabine, we treated noninfected, lv-NC-infected, or lv-DSCR9-infected PANC-1 and MIAPaCa-2 cell lines with gemcitabine (10 μM) for 48 h and examined cell apoptosis. DSCR9 overexpression significantly enhanced gemcitabine-induced effects on the apoptosis of both pancreatic cancer cell lines ( Figure 2F). These data indicated that DSCR9 could inhibit cell proliferation, invasion and gemcitabine resistance in pancreatic cancer cell lines

### DSCR9-related factors and functional analysis of BTG2

**Figure 3:**
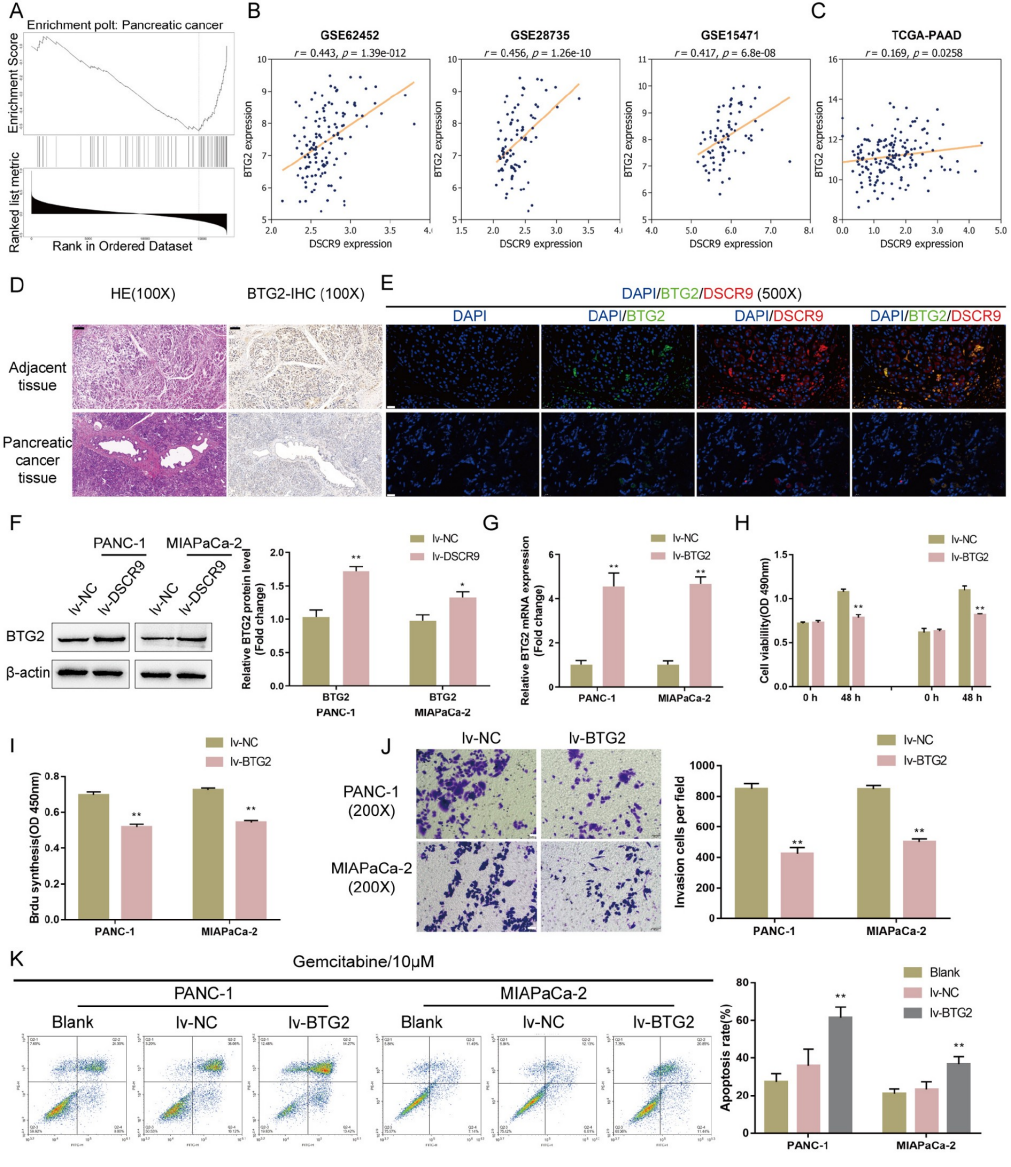
Figure 3 Selection of DSCR9-related factors and functional analysis of BTG2 (A) Subjects from the TCGA-PAAD database were grouped by DSCR9 expression to select differentially expressed factors related to DSCR9 expression, and 212 genes were found to be coexpressed with DSCR9. These genes were applied to Gene Set Enrichment Analysis (GSEA), and they were found to be significantly enriched in hsa05212: pancreatic cancer (FDR=0, P value=0, size=75, number of leading-edge IDs=33, enrichment score=–0.52752, normalized enrichment score=–2.3479). (B) Correlation between BTG2 and DSCR9 expression according to GSE62452, GSE28735, and GSE15471. (C) Correlation between BTG2 and DSCR9 expression according to the TCGA-PAAD database. (D) Histopathological features of pancreatic cancer and noncancerous tissues are shown by H&E staining. The protein content and distribution of BTG2 in tissue samples were shown by IHC staining. (E) The expression levels of lncRNA DSCR9 and the BTG2 protein in pancreatic cancer and adjacent noncancerous tissues were determined by FISH combined with IF staining. Green fluorescence represents the BTG2 level; red fluorescence represents the DSCR9 level. (F) PANC-1 and MIAPaCa-2 cells were infected with lv-DSCR9 and examined for the protein levels of BTG2 by western blot analysis. (G) BTG2 overexpression was generated in PANC-1 and MIAPaCa-2 cells by the transfection of lv-BTG2. The transfection efficiency was confirmed by real-time PCR. Next, PANC-1 and MIAPaCa-2 cells were infected with lv-NC (negative control) or lv-BTG2 and examined for (H) cell viability by CCK-8 assay; (I) DNA synthesis capacity by EdU assay; (J) cell invasion by Transwell assay. (K) Noninfected, lv-NC-infected, or lv-BTG2-infected PANC-1 and MIAPaCa-2 cells were treated with gemcitabine (10 μM) for 48 h and examined for cell apoptosis by flow cytometry. * P<0.05, ** P<0.01 compared to the lv-NC group.

To investigate the mechanism of DSCR9 function in pancreatic cancer cells, we further analyzed online data to select DSCR9-related factors. Subjects from the TCGA database were grouped by DSCR9 expression to select differentially expressed factors related to DSCR9 expression, and 212 genes were found to be coexpressed with DSCR9 (data not shown). These genes were subjected to GeneSet Enrichment Analysis (GSEA), and they were found to be significantly enriched in hsa05212: pancreatic cancer (FDR=0, *P* value=0, size=75, number of leading-edge IDs=33, enrichment score=–0.52752, normalized enrichment score=–2.3479) ( Figure 3A). Among these genes, *BTG2* is underexpressed in pancreatic cancer, while its overexpression suppresses pancreatic cancer cell growth and enhances cancer cell apoptosis [29]. Thus, *BTG2* functions as an antiproliferative gene in tumorigenesis and the effect of DSCR9 in pancreatic cancer cells might be related to BTG2. This speculation was further supported by the online microarray expression profiles GSE62452, GSE28735, and GSE15471 ( Figure 3B); there is a significant positive correlation between DSCR9 and BTG2. Similarly, the TCGA-PAAD database also revealed a positive correlation between DSCR9 and BTG2 ( Figure 3C).

Next, we examined the expression of BTG2 under different conditions. The histopathological features of pancreatic cancer and noncancerous tissue samples were observed by HE staining ( Figure 3D). IHC staining revealed the protein content and distribution of BTG2 in tissue samples; as shown in Figure 3D, the number of BTG2-positive cells was much lower in pancreatic cancer tissues. Moreover, FISH and IF staining results showed that both BTG2 (green fluorescence) and DSCR9 (red fluorescence) levels were upregulated in adjacent tissues compared to those in pancreatic cancer tissues ( Figure 3E). In lv-DSCR9-infected PANC-1 and MIAPaCa-2 cells, BTG2 protein levels were significantly increased ( Figure 3F), confirming the positive correlation between DSCR9 and BTG2.

To validate the specific roles of BTG2 in pancreatic carcinoma cells, we infected PANC-1 and MIAPaCa-2 cells with lv-BTG2 to generate BTG2-overexpressing cells and performed real-time PCR to verify the infection efficiency ( Figure 3G). Next, PANC-1 and MIAPaCa-2 cells were transduced with lv-NC (negative control) or lv-BTG2. Similar to DSCR9 overexpression, BTG2 overexpression significantly inhibited cell viability ( Figure 3H), DNA synthesis capacity ( Figure 3I), and cell invasion ( Figure 3J). Under gemcitabine treatment, BTG2 overexpression also enhanced the promotive effects of gemcitabine on pancreatic cancer cell apoptosis ( Figure 3K).

These data indicated that BTG2 plays anti-tumor roles in pancreatic cancer cells.

### miR-21-5p simultaneously targets DSCR9 and BTG2

**Figure 4:**
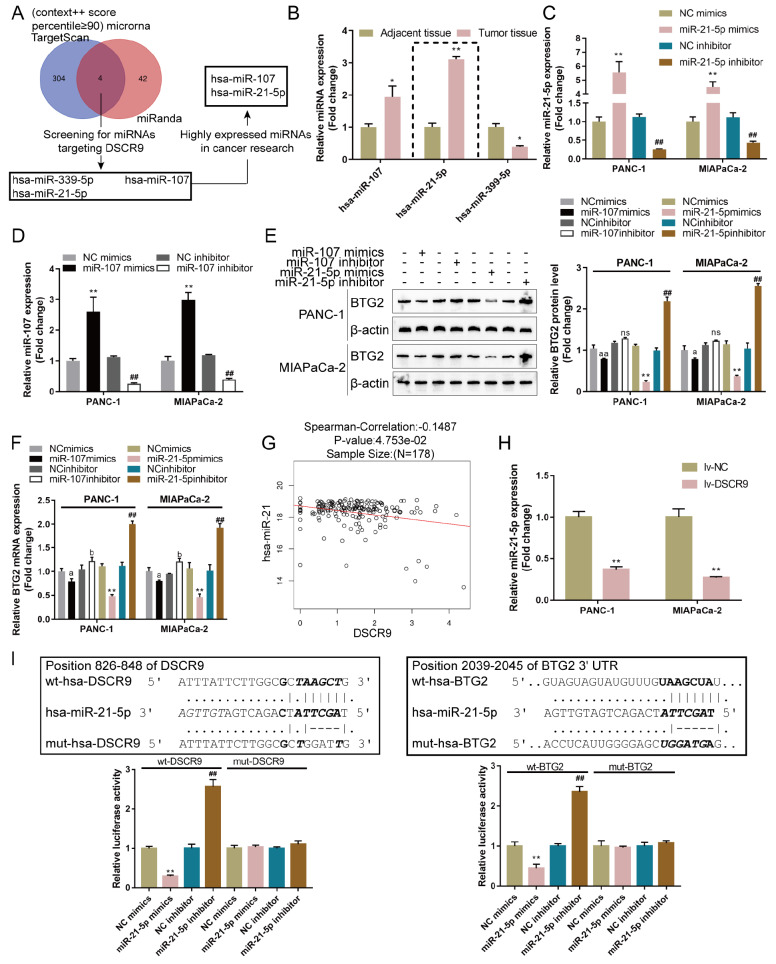
Figure 4 miR-21-5p simultaneously targets DSCR9 and BTG2 (A) The online tools TargetScan and miRanda were used to predict miRNAs that might simultaneously target both DSCR9 and BTG2, and miR-339-5p, miR-107, and miR-21-5p were selected. Among these three miRNAs, miR-107 and miR-21-5p were highly expressed in cancers according to previous studies. (B) The expressions of miR-339-5p, miR-107, and miR-21-5p were examined in pancreatic cancer and noncancerous tissues by real-time PCR. (C,D) The transfection efficiency of miR-21-5p/miR-107 inhibitor or mimics was determined by real-time PCR. (E,F) The expression level of BTG2 in response to miR-21-5p/miR-107 inhibitor or mimics was determined by western blot analysis and real-time PCR. (G) The correlation of miR-21-5p and DSCR9 expression according to the TCGA database. (H) PANC-1 and MIAPaCa-2 cells were infected with lv-DSCR9 and examined for the expression of miR-21-5p by real-time PCR. (I) Wild-type and mutant DSCR9 and BTG2 luciferase reporter vectors were constructed. Mutant-type vectors contained a 4- or 5-bp mutation in the predicted miR-21-5p binding site. These vectors were cotransfected into 293T cells with miR-21-5p mimics/inhibitor, and the luciferase activity was determined. * P<0.05, ** P<0.01, compared to adjacent tissue, NC mimics or lv-NC group; ## P<0.01 compared to NC inhibitor group.

Since miRNAs have been reported to mediate the crosstalk between lncRNAs and mRNAs, we next used the online tools TargetScan and miRanda to predict miRNAs that might simultaneously target both DSCR9 and BTG2. Figure 4A shows that miR-339-5p, miR-107, and miR-21-5p were the possible candidates; among these three miRNAs, miR-107 [ 30, 31] and miR-21-5p [ 32, 33] were highly expressed in cancers according to previous studies. Next, the expression levels of miR-339-5p, miR-107, and miR-21-5p were examined in pancreatic carcinoma and noncancerous tissues, and the results showed that miR-107 and miR-21-5p were dramatically increased, whereas the expression of miR-339-5p was decreased in pancreatic cancer tissue samples ( Figure 4B). After transfection with miR-21-5p mimics, miR-21-5p inhibitor, miR-107 mimics, or miR-107 inhibitor in PANC-1 and MIAPaCa-2 cells ( Figure 4C,D), the level of BTG2 was determined by western blot analysis and real-time PCR. miR-21-5p mimics and miR-107 mimics reduced the level of BTG2, while miR-21-5p inhibitor and miR-107 inhibitor increased the level of BTG2. miR-21-5p was selected for further experiments because it was more effective in modulating BTG2 expression ( Figure 4E,F). Furthermore, the expressions of miR-21-5p and DSCR9 had a negative correlation with each other according to the TCGA database ( Figure 4G). In lv-DSCR9-infected PANC-1 and MIAPaCa-2 cell lines, miR-21-5p expression was significantly downregulated ( Figure 4H).

To investigate the predicted binding of miR-21-5p to DSCR9 and BTG2, we constructed two different DSCR9 and BTG2 luciferase reporter vectors, wild-type and mutant-type. Within the putative miR-21-5p binding site of mutant-type reporter vectors, 4 (mut-DSCR9) or 5 (mut-BTG2) bases were mutated. We cotransfected these vectors into 293T cells with miR-21-5p mimics or inhibitor and examined the luciferase activity. As shown in Figure 4I, wild-type DSCR9 or BTG2 3′UTR luciferase activity was markedly decreased via the overexpression of miR-21-5p but increased via the inhibition of miR-21-5p; mutating the putative miR-21-5p binding site abolished the alterations in luciferase activity. These findings show that miR-21-5p directly binds to DSCR9 and the BTG2 3′UTR.

### Specific roles of miR-21-5p in pancreatic carcinoma cell phenotype

**Figure 5:**
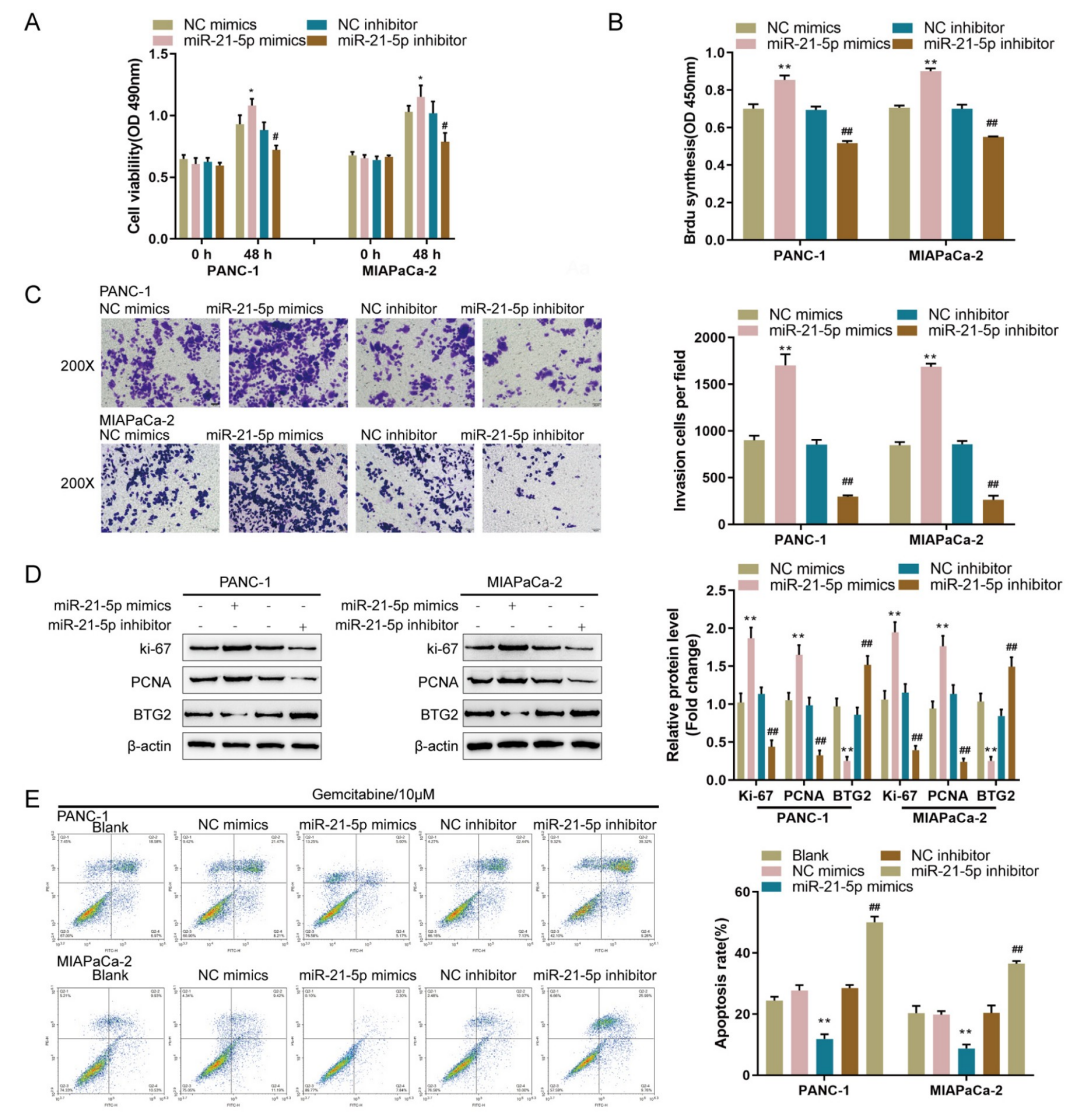
Figure 5 Specific effects of miR-21-5p on pancreatic cancer cell phenotype (A–D) PANC-1 and MIAPaCa-2 cells were transfected with miR-21-5p mimics or miR-21-5p inhibitor. (A) Cell viability by CCK-8 assay. (B) DNA synthesis capacity by EdU assay. (C) Cell invasion by Transwell assay. (D) The protein levels of ki-67, PCNA, and BTG2 by western blot analysis. (E) Cell apoptosis by flow cytometry. Nontransfected, NC mimic/miR-21-5p mimic-transfected, or NC inhibitor/miR-21-5p inhibitor-transfected PANC-1 and MIAPaCa-2 cells were treated with gemcitabine (10 μM) for 48 h and examined for cell apoptosis by flow cytometry. * P<0.05, ** P<0.01, compared to the NC mimics group; # P<0.05, ## P<0.01 compared to the NC inhibitor group.

After confirming the binding of miR-21-5p to DSCR9 and the BTG2 3′UTR, the specific roles of miR-21-5p in the pancreatic carcinoma phenotype were validated. We assigned PANC-1 and MIAPaCa-2 cell lines into four groups: transfection with NC mimics group (negative control), transfection with miR-21-5p mimics group, transfection with NC inhibitor group (negative control), and transfection with miR-21-5p inhibitor group. Within both cell lines, overexpression of miR-21-5p significantly enhanced cell viability, DNA synthesis capacity, and cell invasion, whereas inhibition of miR-21-5p had the opposite effect ( Figure 5A‒C). Overexpression of miR-21-5p consistently increased, while inhibition of miR-21-5p decreased, the ki-67 and PCNA protein contents ( Figure 5D). Under gemcitabine treatment, overexpression of miR-21-5p inhibited apoptosis, while the inhibition of miR-21-5p promoted apoptosis in both pancreatic cancer cell lines ( Figure 5E). These results indicate that miR-21-5p is carcinogenic in pancreatic cancer cell lines.

### Dynamic effects of DSCR9 and miR-21-5p on BTG2 and pancreatic carcinoma cell phenotypes

**Figure 6:**
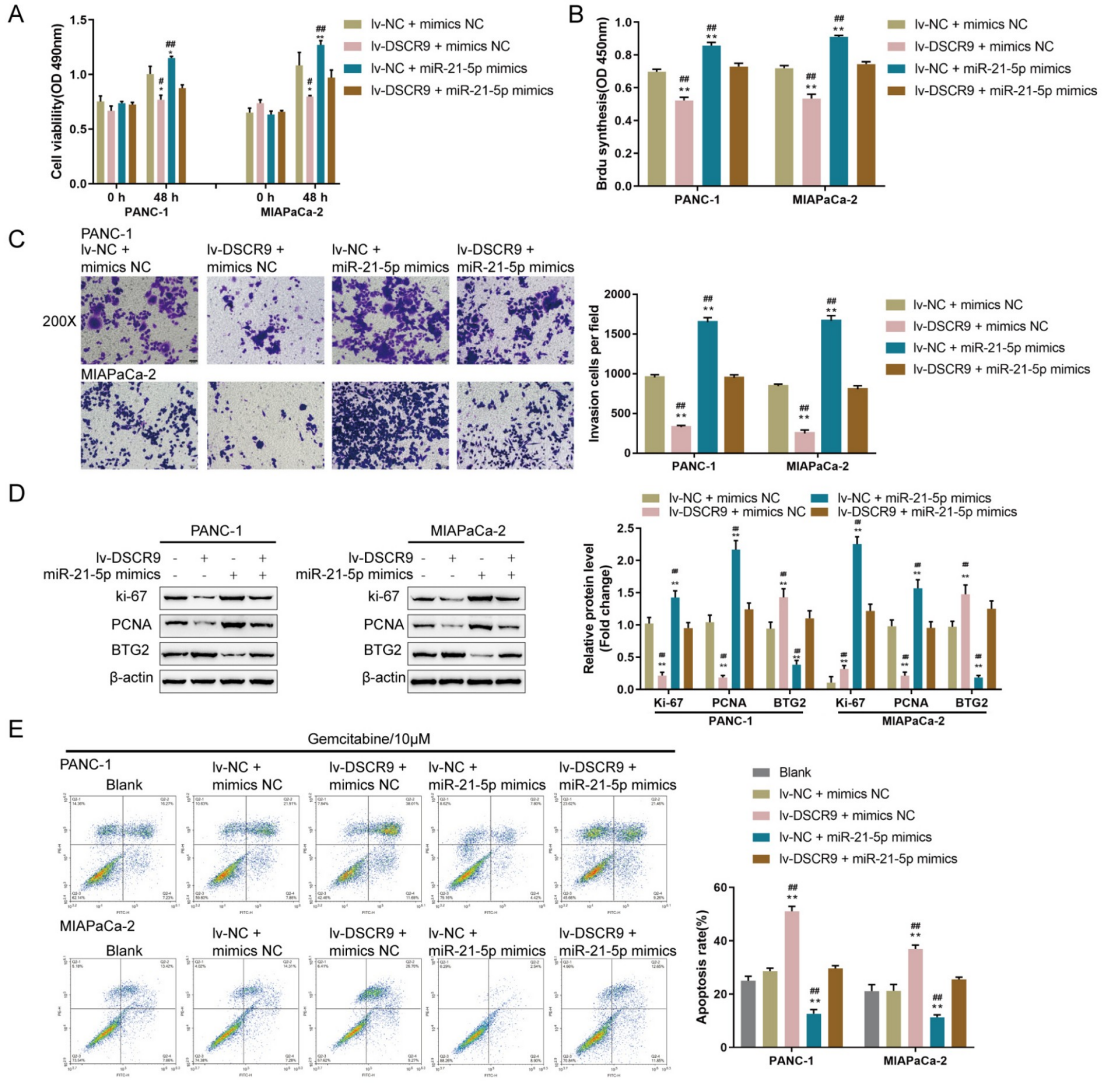
Figure 6 Dynamic effects of DSCR9 and miR-21-5p on BTG2 and pancreatic cancer cell phenotypes (A–D) PANC-1 and MIAPaCa-2 cells were cotransfected with lv-DSCR9 and miR-21-5p mimics, and sub. (A) Cell viability by CCK-8 assay. (B) DNA synthesis capacity by EdU assay. (C) Cell invasion by Transwell assay. (D) the protein levels of ki-67, PCNA, and BTG2 by western blot analysis. (E) Cell apoptosis by flow cytometry. PANC-1 and MIAPaCa-2 cells were untransfected or cotransfected with lv-DSCR9 and miR-21-5p mimics under gemcitabine treatment (10 μM) for 48 h and examined for cell apoptosis by flow cytometry. * P<0.05, ** P<0.01, compared to the control group; # P<0.05, ## P<0.01, compared to the lv-DSCR9+mimics NC or lv-NC+miR-21-5p mimics group.

Since miR-21-5p directly targets DSCR9 and the BTG2 3′UTR, the dynamic effects of DSCR9 and miR-21-5p on BTG2 expression and pancreatic cancer cell phenotype were examined to determine whether DSCR9 and miR-21-5p exert their functions via BTG2. PANC-1 and MIAPaCa-2 cells were assigned to four groups: transfection of lv-NC+mimics NC group (negative control), transfection of lv-DSCR9+mimics NC group, transfection of lv-NC+miR-21-5p mimics group, and transfection of lv-DSCR9+miR-21-5p mimics group. In both pancreatic cancer cell lines, cell viability, DNA synthesis capacity, and cell invasion were markedly decreased by DSCR9 overexpression but increased by miR-21-5p overexpression; the effects of DSCR9 overexpression on pancreatic cancer phenotype were significantly reversed by miR-21-5p overexpression ( Figure 6A‒C). Consistently, DSCR9 overexpression inhibited, while miR-21-5p overexpression promoted, ki-67 and PCNA protein levels; moreover, the effects of DSCR9 overexpression on these two proteins were significantly reversed by miR-21-5p overexpression ( Figure 6D). Under gemcitabine treatment, DSCR9 overexpression increased, whereas miR-21-5p overexpression decreased cell apoptosis; miR-21-5p overexpression significantly reversed the effects of DSCR9 overexpression on pancreatic cancer cell apoptosis ( Figure 6E). More importantly, DSCR9 overexpression increased the BTG2 protein content, while miR-21-5p overexpression decreased it; miR-21-5p overexpression dramatically reversed the effects of DSCR9 overexpression on the BTG2 protein content ( Figure 6D). Thus, the DSCR9/miR-21-5p axis regulates the proliferation, invasion, and gemcitabine resistance of pancreatic carcinoma cells via BTG2.

### miR-21-5p and BTG2 expressions and their correlation within tissues

**Figure 7:**
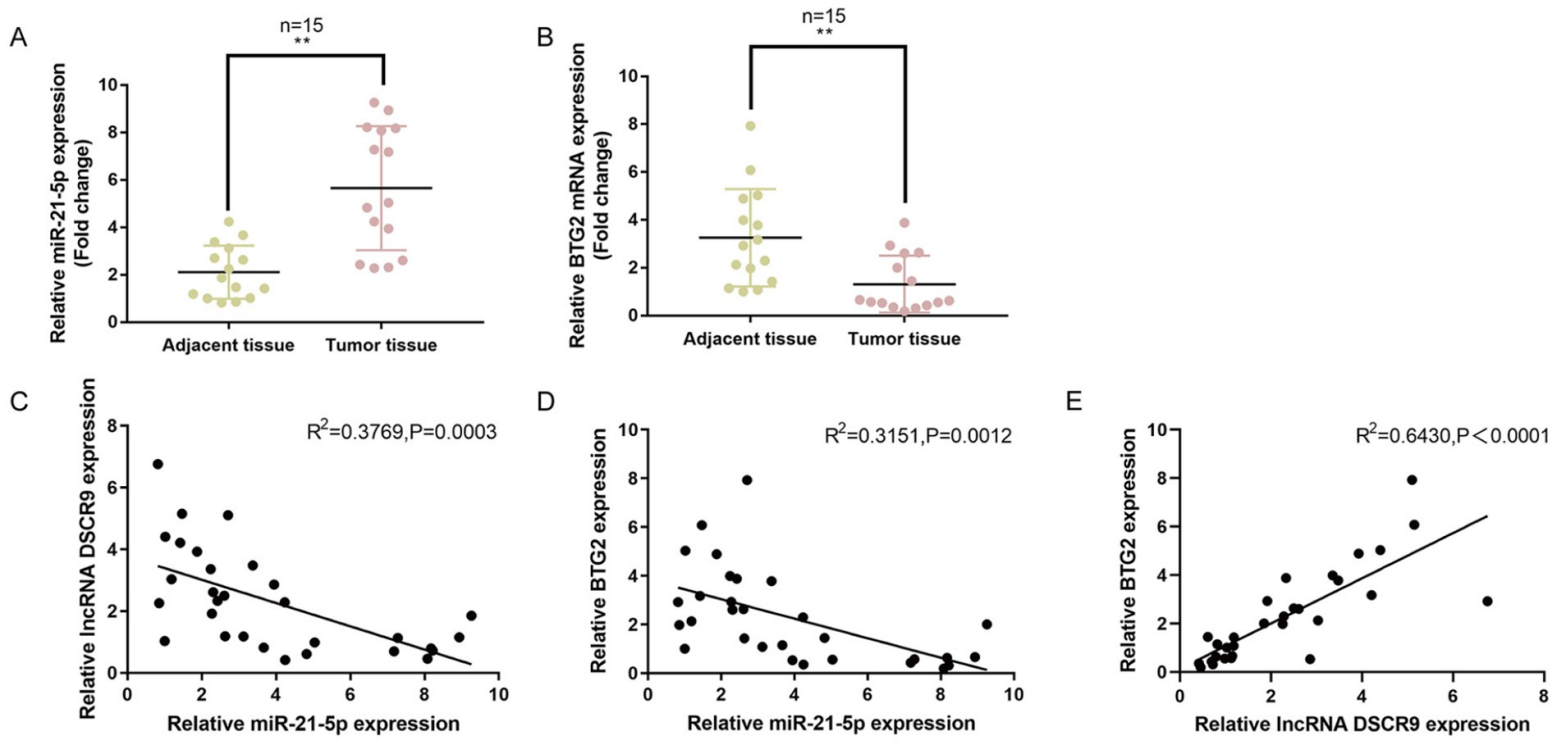
Figure 7 Expression and correlation of miR-21-5p and BTG2 in tissue samples (A,B) The expressions of miR-21-5p and BTG2 were determined in 15 paired pancreatic cancer and noncancerous tissue samples by real-time PCR. (C–E) Pearson’s correlation analysis was used to analyze the correlation between miR-21-5p and DSCR9 (C), miR-21-5p and BTG2 (D), and DSCR9 and BTG2 (E). ** P<0.01.

As a further confirmation, we examined miR-21-5p and BTG2 expression in 15 paired pancreatic cancer and noncancerous tissue samples. As shown in Figure 7A,B, the expression of miR-21-5p was dramatically increased, whereas the expression of BTG2 was decreased in pancreatic cancer tissue samples compared with those in noncancerous tissues. As analyzed by Pearson′s correlation analysis, miR-21-5p had a negative correlation with DSCR9 and BTG2. In addition, DSCR9 had a positive correlation with BTG2 ( Figure 7C‒E). These data indicated that the DSCR9/miR-21-5p/BGT2 axis may exist in pancreatic cancer.

## Discussion

Here, we demonstrate that the lncRNA DSCR9 is an important factor associated with the prognosis of pancreatic cancer patients. The lncRNA DSCR9 showed a significant reduction in pancreatic cancer tissue samples and cells. DSCR9 overexpression significantly inhibited the proliferation and invasion of pancreatic cancer cells while enhancing apoptosis under gemcitabine treatment. A positive correlation between BTG2 expression and DSCR9 was observed. BTG2 also exerted tumor-suppressive effects by suppressing the proliferation and invasive ability of pancreatic cancer cells and increasing gemcitabine-stimulated apoptosis. miR-21-5p targeted both DSCR9 and BTG2. DSCR9 negatively regulated miR-21-5p expression, while miR-21-5p negatively regulated BTG2 expression. In contrast to DSCR9 overexpression, miR-21-5p overexpression enhanced the aggressiveness of pancreatic cancer cells by promoting the proliferation and invasive ability of cancer cells and attenuating gemcitabine-stimulated apoptosis. More importantly, the tumor-suppressive effects of DSCR9 overexpression on pancreatic cancer cells were significantly reversed by miR-21-5p overexpression, whereas the suppressive effects of DSCR9 overexpression on BTG2 expression were reversed by miR-21-5p overexpression. Finally, the expression of miR-21-5p was elevated in pancreatic cancer tissue samples, while BTG2 was reduced. In addition, miR-21-5p was negatively correlated with DSCR9 and BTG2.

lncRNA DSCR9 is transcribed from the Down syndrome critical region (DSCR) of chromosome 21 [34]. When DSCR9 was first identified, 14 genes that were coexpressed with DSCR9 were reported to function in EGFR signaling by correlation-interaction-network (COIN) analysis [35]. Additionally, according to the COIN analysis results, the SRC gene was regarded as the most prominent gene target candidate within the DSCR9 regulatory network [35]. The proto-oncogene tyrosine-protein kinase Src is a nonreceptor tyrosine kinase protein that in humans is encoded by the SRC gene, and the Src pathway is active within approximately fifty percent of tumors in the colon, liver, lung, breast, and pancreas [36]. Another key regulatory target of DSCR9 within the identified network was EGFR, which was recognized as a widely studied oncogene and an important drug target related to multiple anticancer therapeutics, including gefitinib, erlotinib, afatinib, brigatinib, and icotinib for pulmonary carcinoma and cetuximab for colon carcinoma. Herein, the expression of DSCR9 was markedly decreased in pancreatic carcinoma tissue samples and cell lines. Forced overexpression of DSCR9 in pancreatic cancer cells inhibited the proliferation and invasive ability of cancer cells while enhancing gemcitabine-induced apoptosis, suggesting that DSCR9 has a tumor-suppressive effect on pancreatic cancer.

To further investigate the molecular mechanism, we also analyzed the genes coexpressed with DSCR9 using an online database. Notably, DSCR9 expression was significantly positively correlated with BTG2 expression in tissue samples. As we have mentioned, BTG2 exerts a tumor inhibitory effect on various cancers, such as pancreatic carcinoma. BTG2 expression was found to be significantly reduced in PAAD [37]. BTG2 plasmid transduction attenuates the capacity of pancreatic cancer cells to proliferate and invade while enhancing cell apoptosis [29]. Furthermore, we observed that *BTG2* knockdown in pancreatic cancer cell lines dramatically increased the capacity of cancer cells to proliferate and invade while reducing gemcitabine-induced apoptosis. Consistent with the bioinformatics analysis results, DSCR9 overexpression caused a remarkable increase in the expression level of BTG2, suggesting that DSCR9 positively regulates BTG2. Since BTG2 has been reported to be targeted by several miRNAs within pancreatic carcinomas, such as miR-21 [37] and miR-27a [29], we speculate that there might be other miRNAs mediating the crosstalk between DSCR9 and BTG2 in pancreatic cancer cells. Through online tool prediction and experimental validation, we revealed that miR-21-5p targeted DSCR9 and BTG2. DSCR9 negatively regulated miR-21-5p expression, while miR-21-5p was targeted to suppress BTG2 expression.

Previously, miR-21-5p was regarded as a strong prognostic biomarker for cancers. Within young gastric cancer patients with tumors containing a high intratumoral stroma ratio, miR-21-5p could be used to predict recurrence [38]. Within non-small cell lung cancer, miR-21-5p inhibition enhanced cancer cell radiation sensitivity [39], while miR-21-5p overexpression promoted cell invasion and migration by targeting SMAD7 [40]. As one of the most abnormally expressed miRNAs in pancreatic cancer, circulating hsa-miR-21-5p has been identified as a potential biomarker in serum within pancreatic cancer patients [41]. Herein, we performed functional experiments to validate the specific roles of miR-21-5p in pancreatic cancer aggressiveness and drug resistance. Consistent with its functions in other cancers, miR-21-5p overexpression significantly promoted the capacity of pancreatic cancer cells to proliferate and invade while attenuating gemcitabine-induced cell apoptosis. More importantly, when being cotransfected into pancreatic cancer cells with lentivirus overexpressing DSCR9, miR-21-5p mimics significantly attenuated the effects of DSCR9 overexpression on BTG2 protein level, pancreatic cancer cell aggressiveness, and gemcitabine resistance, indicating that the DSCR9/miR-21-5p axis modulates pancreatic cancer cell proliferation, invasion, and drug resistance through BTG2. As a further confirmation of these *in vitro* findings, miR-21-5p expression was significantly increased, while BTG2 expression was decreased in pancreatic carcinoma tissues compared with those in noncancerous tissues. miR-21-5p had a negative correlation with DSCR9 and BTG2.

In conclusion, we demonstrated that the lncRNA DSCR9/miR-21-5p/BTG2 axis modulates pancreatic cancer proliferation, invasion, and resistance to gemcitabine. Although gemcitabine monotherapy remains the gold standard of treatment for advanced PAAD, gemcitabine combined with nab-paclitaxel or FOLFIRINOX has emerged as a new strategy of the treatment for patients with metastatic pancreatic cancer. The functions of the lncRNA DSCR9/miR-21-5p/BTG2 axis in new pancreatic cancer treatment strategies await further investigation.

## Supporting information

243FigS1-S2_TableS

083Table1

083Table2

## Supplementary Data

Supplementary data is available at *Acta Biochimica et Biphysica Sinica* online.
