# Supplementary material for: DSCR9/miR-21-5p axis inhibits pancreatic cancer proliferation and resistance to gemcitabine via BTG2 signaling: DSCR9/miR-21-5p/BTG2 regulates pancreatic cancer
Source: Acta Biochim Biophys Sin (Shanghai). 2022 Dec 26;54(12):1775–88. doi: 10.3724/abbs.2022194 (PMC10157615; doi:10.3724/abbs.2022194)
Supplement: 243FigS1-S2_TableS [file 243FigS1-S2_TableS.pdf]

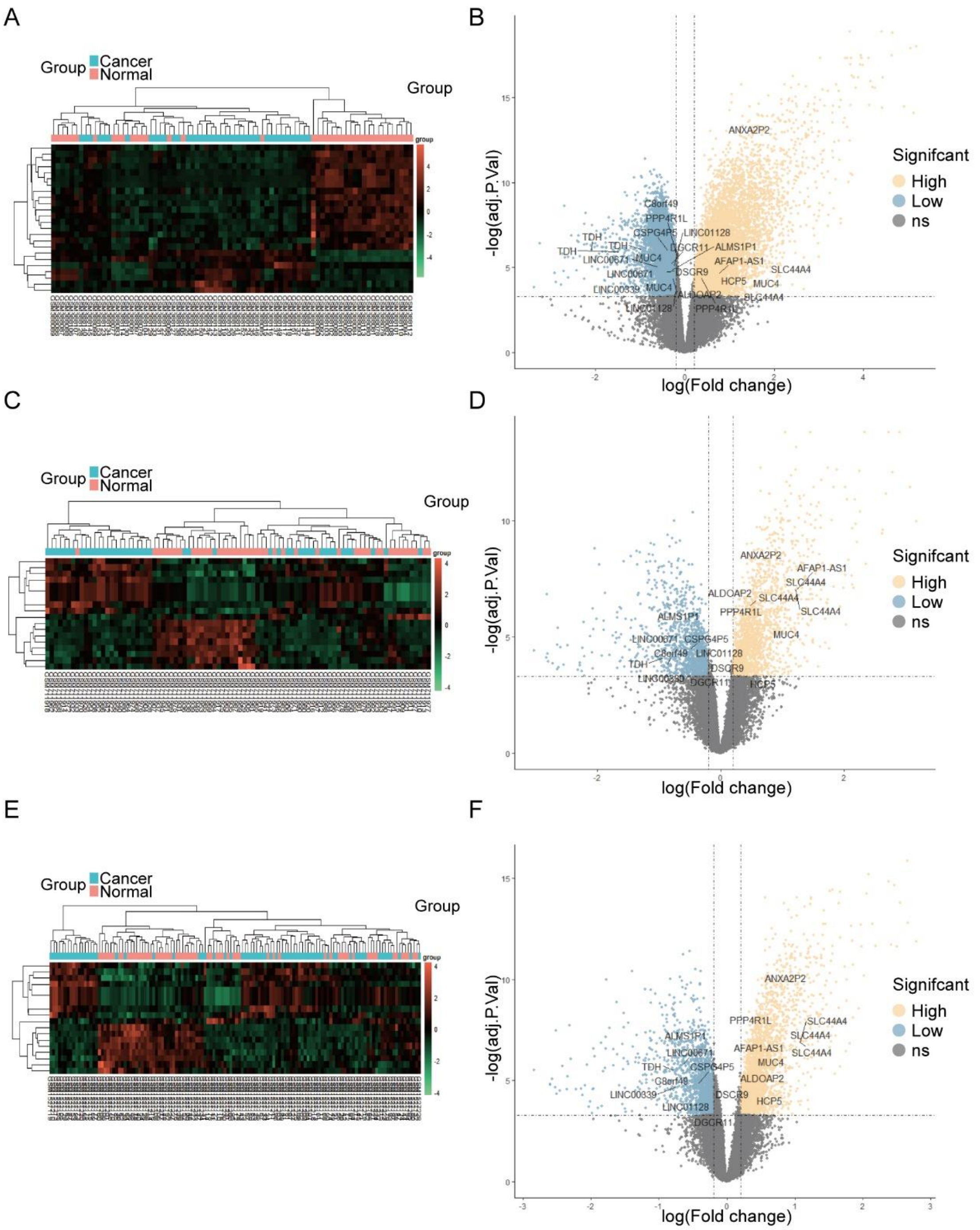

Supplementary Figure S1. Bioinformatics analysis of pancreatic cancer chips from the Gene Expression Omnibus (GEO) (A,B) GSE15471. (C,D) GSE62452. (E,F) GSE28735.

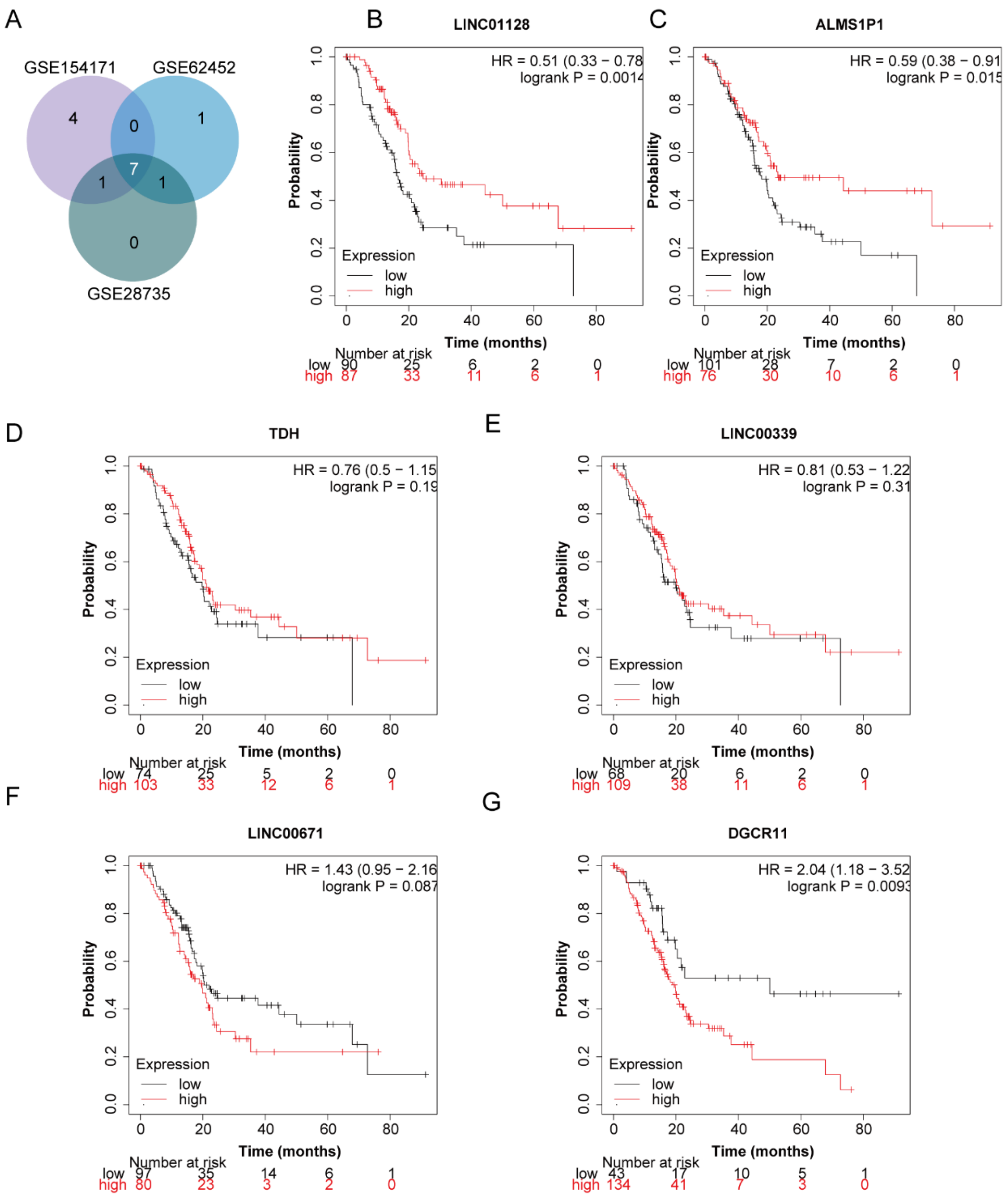

Supplementary Figure S2. The association between the expression of lncRNA and pancreatic carcinoma patients' survival rate in Kmplot

Supplementary Table S1. The clinical character of patients

No.	Age	Gender	Tumor location	TNM	Stage
1	54	F	Head	T2N0M0	IB
2	68	F	Head	T1N0M0	IA
3	70	F	Head	T3N0M0	IIA
4	53	M	Head	T1N0M0	IA
5	64	M	Head	T2N1M0	IIB
6	50	M	Head	T1N1M0	IIB
7	66	F	Head	T1N0M0	IA
8	57	F	Head	T1N1M0	IIB
9	38	F	Head	T1N0M0	IA
10	67	M	Head	T2N0M0	IB
11	70	F	Head	T2N0M0	IB
12	65	M	Head	T1N1M0	IIB
13	75	F	Head	T1N0M0	IA
14	56	M	Head	T1N0M0	IA
15	53	M	Head	T2N0M0	IB
